# Gambian Mothers Lack Obstetric Danger Sign Knowledge, But Educational Intervention Shows Promise

**DOI:** 10.5334/aogh.3930

**Published:** 2024-05-20

**Authors:** Kara Shannon, Jocelyn Burridge, Brodus Franklin, Sheena Bhushan, Susan Hilsenbeck, Elena V. Petrova, James N’Dow, Ibezimako Iwuh, Sharmila Anandasabapathy, Jeffrey P. Wilkinson

**Affiliations:** 1 School of Medicine, Baylor College of Medicine, Houston, TX, USA; 2 Baylor Global Health, Houston, TX, USA; 3 Dan L. Duncan Comprehensive Cancer Center, Baylor College of Medicine, Houston, TX, USA; 4 Horizons Trust Gambia, The Gambia; 5 Academic Urology Unit, Institute of Applied Health Sciences, University of Aberdeen, Aberdeen, UK; 6 Department of Obstetrics and Gynecology, Baylor College of Medicine, Houston, TX, USA; 7 Global Women’s Health, Department of Obstetrics and Gynecology, Baylor College of Medicine, Houston, TX, USA

**Keywords:** complications, obstetric labor, global health, health literacy, complication, pregnancy complication, labor, educational status, maternal care patterns, maternal child health center, maternal educational status

## Abstract

**Background:**

The Gambia has the 12^th^ highest maternal mortality rate in the world, with 80% of deaths resulting from avoidable causes. Unawareness of pregnancy danger signs (DS) has been shown to be a barrier to seeking obstetric care, while app-based education intervention has shown promise.

**Objective:**

We aim to assess patient awareness of DS, identify barriers to awareness, and evaluate potential for implementing smartphone-based technologies for education.

**Methods:**

A cross-sectional semi-structured survey was administered to Gambian women (n = 100) across five hospitals/health centers. Data and informed consent were collected via an online survey portal. Analysis included bivariate analysis and descriptive statistics with p < 0.05 significance level. Recall of 0–2 DS per category was classified as “low” knowledge, 3–5 as “moderate” knowledge, and 6+ as “sufficient” knowledge. Cross-category recall was quantified for overall awareness level (0–6 = “low”, 7–12 = “moderate”, 13+ = “sufficient”. N = 28 total DS).

**Findings:**

Although 75% of participants (n = 100) self-perceived “sufficient” knowledge of DS, the average recall was only two (SD = 2, n = 11) pregnancy DS, one labor and delivery DS (SD = 1, n = 8), and one postpartum DS (SD = 1, n = 9). Twenty-one women were unable to recall any danger signs. “Low” awareness was identified in 77% of women, while 23%, and 0% of women showed “moderate” and “sufficient” overall awareness, respectively. Education level was significantly correlated with overall danger sign recall (ρ(98) = .243, p = .015) and awareness level (ρ(98) = .265, p = .008). Monthly income was significantly correlated with awareness level (ρ(97) = .311, p = .002). Smartphone ownership was reported by 76% of women, and 97% expressed interest in using app-based video (94%) or provider (93%) teaching.

**Conclusions:**

Women had low knowledge of obstetric danger signs, and true awareness of danger signs was remarkably lower than self-perceived knowledge. However, patients exhibited proper healthcare-seeking behavior when danger signs arose. Findings suggest that video- or messaging-based education from local healthcare providers may be effective DS educational interventions.

## Introduction

Maternal mortality remains an immense burden on society and a painful reality for women and their families, especially in low-income countries. In 2019, the WHO released a consensus statement and strategy paper on ending preventable maternal mortality, targeting a global maternal mortality rate of less than 70 per 100,000 live births by the year 2030 [1]. However, progress towards the goal has been slow. In the year 2017, there were 295,000 maternal deaths globally, with an average of 211 maternal deaths per 100,000 live births. Many of these fatalities occurred in low-income countries, with Sub-Saharan Africa alone accounting for 66% of global maternal deaths, with an estimate of 542 maternal deaths per 100,000 live births. In stark contrast, high-income countries such as Australia and New Zealand have maternal mortality rates of 7 per 100,000 live births–almost 70 times lower than in many African countries [1,2].

The Gambia, a small country located on the western coast of Africa, experiences this challenge. The country has the 12^th^ highest maternal mortality rate in the world, with the most recent documented estimate in 2017 of 597 maternal deaths per 100,000 live births. Maternal mortality rates in The Gambia are trending in the right direction with a 36% reduction in maternal mortality rate between 2000 and 2017 [1]. Nonetheless, efforts towards making necessary interventions to drastically reduce maternal mortality rates have been insufficient [3]. It is estimated that 80% of maternal deaths in low-income countries result from obstetric causes such as hemorrhage, obstructed labor, sepsis, and pre-eclampsia/eclampsia [4]. These conditions are many times preventable or easily treatable, requiring little or no extra cost even in low-income countries with minimal infrastructure and resources [2].

The SmartPod-based Labor and Delivery Intervention and Training program in The Gambia, implemented by *Baylor Global Health* in collaboration with Horizons Trust Gambia, seeks to address and understand provider deficits and associated factors that contribute to the high maternal mortality rate. The goal is to use this insight to guide the development of future programs and interventions to reduce the number of preventable maternal and neonatal deaths.

However, this is only one piece of the puzzle. While severe capacity limitations (e.g., inadequate infrastructure, poor access to skilled obstetrics providers, and untimely access to high-quality care including cesarean delivery) contribute to high mortality rates in under-resourced countries, studies in countries such as Tanzania, Ghana, the Democratic Republic of Congo, and Ethiopia identified lack of adequate awareness by pregnant women about pregnancy danger signs and complications as a major barrier to seeking timely obstetric care. These studies also demonstrated that delayed obstetric care after the presentation of high-risk conditions is correlated with markedly reduced survival rates among mothers [5,6,7,8,9,10].

Qualitative analyses to assess maternal awareness of these danger signs via questionnaires have been carried out in other countries such as Ethiopia and Tanzania. In these studies, it was found that a large proportion of pregnant mothers lack knowledge of obstetric danger signs [5,6,7]. Following education on these topics, pregnant mothers sought appropriate medical attention upon recognizing obstetric danger signs during pregnancy. The majority, however, had low knowledge of these signs at baseline, posing a barrier to timely and appropriate obstetric care [5,11,12]. To date, no similar patient awareness studies have been performed in The Gambia.

Study findings in other African countries suggest that augmenting awareness in places of knowledge deficiencies could increase proactivity of seeking medical care with the intention of decreasing maternal mortality [6,7,13]. Low knowledge of danger signs among pregnant mothers in The Gambia may contribute to the high maternal mortality rate and may be an ideal target for interventions like those implemented in other countries. An assessment in rural South Africa demonstrated that the majority of women own mobile smartphones, and proposed that a technology-based solution for teaching women about these warning signs of compromised maternal or fetal health during pregnancy would be beneficial [14]. Similar technology-based interventions for obstetric complication awareness have been suggested for use in Uganda, and the methods have already shown efficacy for HIV-related educational interventions [15,16,17]. Smartphone or technology-based education tools are a scalable and cost-effective approach that could be implemented as a long-term education platform to increase awareness of obstetric danger signs among pregnant mothers.

Although public awareness of obstetric danger signs and associated resources for maternal healthcare services are essential to improving maternal mortality, little is known about patient awareness in The Gambia. Therefore, it is difficult to gauge which methods of education and targeted intervention would be most effective. The aims of this study are two-fold: (1) to assess and identify barriers to patient awareness of obstetric danger signs during pregnancy, labor and delivery, and the postpartum period; and (2) to evaluate the feasibility and attitudes of Gambian mothers towards the implementation of smartphone-based interventions for education regarding danger signs.

## Methods

### Study Design

This study is a community based cross-sectional study conducted among women in The Gambia. Data were gathered at one time point. Approved was granted by the Institutional Review Board, protocol H-47359.

### Study Setting

This study was performed across four healthcare centers in Serrekunda and Brikama regions of The Gambia: Edward Francis Small Teaching Hospital, Kanifing General Hospital, Brikama District Hospital, and Serrekunda Health Center. These regions have a combined population of approximately 1.1 million [18]. Interviews and data collection were completed over a one-month period during May 2021.

### Study Participants

Eligible participants were women visiting the hospitals/health centers and who had been pregnant at least once within the past five years. One-hundred available and eligible participants were interviewed at random. Participation was anonymous and voluntary, and patient consent was obtained before partaking in the study. Participants were free to withdraw their participation at any time.

### Survey Development

To survey participants, we developed and utilized a semi-structured questionnaire. This medium was based on the “Safe Motherhood Questionnaire” previously designed and implemented by the Maternal Neonatal Program of JHPIEGO [19]. The survey was modified to include danger signs noted in the WHO Guide for Essential Practice (Pregnancy, Childbirth, Postpartum) [20] (Table 1). The assessment questionnaire encompassed six domains: (1) seven questions on sociodemographic factors that may contribute to knowledge and healthcare-seeking behaviors; (2) 12 questions on barriers to healthcare access; (3) 22 questions on recent pregnancy history; (4) ten questions on knowledge of danger signs during pregnancy, childbirth, and the postpartum period; (5) 31 questions on quality of care during pregnancy, delivery, and postpartum and assessment of rapport/trust built by providers with the surveyed women; and (6) 13 questions on technology access and usage opinions in an effort to understand which means of education (technological, instructional, written) would be well-received in the context of local practice. Our questionnaire was developed in English and reverse-translated to the native language by a local midwife and healthcare partner who are native speakers and was tested and revised prior to implementation.

**Table 1:** Obstetric danger signs included in questionnaire, based on the WHO Guide for Essential Practice.


PREGNANCY	LABOR AND DELIVERY	POSTPARTUM (<48H AFTER DELIVERY)

Vaginal Bleeding Vomiting Severe Abdominal Pain Swelling of Face or Legs Blurry Vision No Fetal Movement High Blood Pressure Convulsions (seizure) Severe Headache High Fever Difficulty Breathing	Heavy Bleeding Vaginal Bleeding Labor for >12 hours Abnormal Fetal Position Shoulder Dystocia Severe Headache Retained Placenta High Fever	Vaginal Bleeding Abdominal Pain Blurred Vision High Fever Too Weak to Get Out of Bed Smelly Vaginal Discharge Fast or Difficult Breathing Convulsions Breasts Swollen, Red, or Sore


The section for “assessment of danger signs” was guided by studies in Tanzania by Pembe et al. and Mwilike et al. and are listed in Table 1. Each subset (pregnancy, childbirth, and postpartum) included 5-point Likert scale question such *as* “*I believe that I have sufficient knowledge of the danger signs of health problems that can occur during pregnancy*,” followed by an open-ended question where women were asked to recall danger signs of which they were aware.

The section for barriers to healthcare access included 12 “*yes*” or “*no*” questions surrounding difficulties that may be faced by women when seeking both routine and emergent obstetric care. Sections for “quality of care” and “assessment of support and rapport with providers” included questions to assess quality and quantity of education given by providers (doctors, midwives, nurses, traditional birth attendants), and trust in providers. We also surveyed women about the origin of most of their pregnancy-related education and included questions on healthcare-seeking behavior and action in emergent situations. The section on technology access and attitudes was guided by similar studies by Coleman and Musiimenta et al., and surveyed women regarding ownership of mobile phones, usage of mobile phones, familiarity with smartphone apps, and preferences regarding education dissemination methods.

### Survey Administration and Data Collection

We had initially planned to administer surveys in-person. However, due to COVID-19, administration was by local Horizons Trust Gambia partners and three midwives who had been trained in administration by our team. Responses were input from tablets and collected via an online survey portal in real-time. Each patient encounter included consent, survey administration, and data collection, all of which lasted approximately 30 minutes. Upon completion of the survey, participants were compensated with mobile phone credit worth D200, the equivalent of four hours paid minimum wage (hourly = D50, equivalent $1.25) [21]. Patients were informed of compensation prior to consent.

### Data Analysis

Descriptive, bivariate, and inferential statistics were calculated using SPSS software (version 28.0; SPSS Inc, Chicago, IL). Classification of awareness of danger signs was based on spontaneous recall during questionnaire administration. Recall of 0–2 signs per category was classified as “low” knowledge, 3–5 as “moderate” knowledge, and 6+ as “sufficient” knowledge. Category-specific danger signal recall scores were summed to obtain an overall recall score (possible range 0–28), which was trichotomized: 0–6 signs was classified as low awareness, 7–12 as moderate awareness, and 13 or more as sufficient awareness of danger signs. Median, IQR, Odds Ratio with 95% confidence interval, and Spearman’s coefficient with a p < 0.05 significance level were calculated. Factors independently controlled for were age, education, monthly income, and gravida status (total number of pregnancies).

## Results

### Sociodemographic characteristics

Sociodemographic characteristics and factors related to most recent pregnancy are summarized in Table 2. All participants were between the ages of 18–49, and the majority of women were married and earned a household monthly income of less than D10,000 which is equal to approximately $180 USD [22].

**Table 2:** Sociodemographic characteristics and factors related to most recent pregnancy of surveyed women (n = 100).


		TOTAL (n)

**Nationality**	Gambian Citizen	91

	Non-Gambian Citizen	8

**Age**	<18	0

	18–24	25

	25–29	28

	30–39	39

	40–49	8

	>50	0

**Marital Status**	Single, never married	4

	Married	92

	Divorced	3

	Widowed	1

**Education Level**	Do not read or write	13

	Less than high school	33

	Completed high school	15

	Arabic School	22

	College	17

	Professional School	7

	Graduate School	1

**Monthly Income**	Less than D 5,000	27

	D 5,000–D 10,000	59

	D 10,001–D 15,000	9

	More than D 15,000	4

**Residence**	Urban	72

	Rural	28

**Gravidity**	G1	19

	G2	13

	G3	19

	G4	18

	G5	14

	G > 5	17

**Delivery Location**	Hospital	56

	Health Center	34

	At home with healthcare supervision	6

	Traditional Birth Attendant	3

	At home unsupervised	1

**Delivery Method**	Vaginal Delivery	74

	Elective C-Section	25

	Emergency C-Section	1


### Barriers to healthcare access

When assessing barriers to receiving obstetric care, participants were asked about experiencing obstacles related to time, location, and finances, the results of which are detailed in Table 3. An overwhelming 91% of respondents reported lack of health insurance or referral as a barrier to care, 56% reported that the health care facility was too far away, and 42% were unable to afford the appointment cost or taxi. It is important to note that of our participant sample, 100% of women noted that they had familial or partner support for attending regular obstetric checkups.

**Table 3:** Reported barriers to healthcare access reported by women, and percentage of women experiencing barrier.


REPORTED BARRIERS TO HEALTHCARE ACCESS	PERCENTAGE OF PARTICIPANTS EXPERIENCING BARRIER

Time	7%

Location (Healthcare facility too far away)	56%

Lack of transportation method	20%

Lack of money for taxi	26%

Lack of health insurance/referral	91%

Inability to afford the obstetrics visit	16%


### Obstetric education and provider relationship

Most participants (78%) received education about pregnancy and childbirth from their provider or through group educational workshops. A similar amount (76%) reported receiving maternal health counseling. Counseling with respect to pregnancy and childbirth was primarily provided by nurses (59.6%), midwives (56.4%), family members (48.9%), and friends (26.6%). Only 19.1% of women reported receiving maternal health counseling from a physician. Furthermore, 81% of participants felt supported by their healthcare provider, and 94% of them reported trusting their healthcare provider to explain their condition, give instruction on medication, and listen to their concerns. Additionally, 98% of women reported trust for western medicine.

### Knowledge of obstetric danger signs

**Figure 1:**
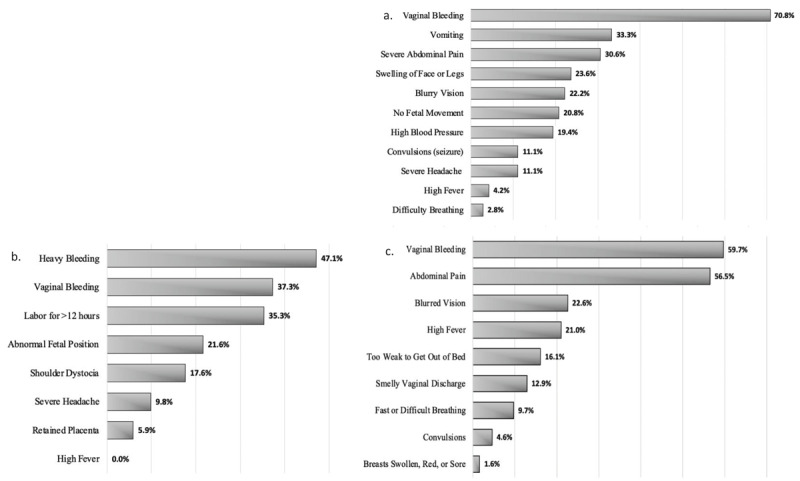
Recall of danger signs during **(a.)** pregnancy (n = 100), **(b.)** labor and delivery (n = 100), **(c.)** postpartum (n = 100).

Eighty-seven percent (87%) of respondents (women) reported having knowledge regarding the potential complications during pregnancy and childbirth. Seventy-five percent (75%) of respondents reported “*agree*” or “*strongly agree*” to the statement, “*I have sufficient knowledge of danger signs during pregnancy*.” Regarding the same statement concerning danger signs during pregnancy, 22% of respondents reported “*disagree*,” 1% “*strongly disagree*,” and 2% “*cannot say*”. However, when respondents were asked to list as many danger signs of pregnancy as possible, the median number listed was 2 (IQR (0–3)) out of 11 possible danger signs. The most registered danger sign for pregnancy was “vaginal bleeding” (Figure 1a).

Fifty-five percent (55%) of respondents reported “*agree*” or “*strongly agree*” to the statement, “*I have sufficient knowledge of the danger signs that can occur during labor and delivery*.” However, 36% of respondents reported “*disagree*,” 5% “*strongly disagree*,” and 4% “*cannot say*” to the same statement concerning danger signs during labor and delivery. The median number of signs listed for labor and delivery was 1 (IQR (0–2)) out of 8 possible. The most registered signs for labor and delivery were “heavy bleeding” and “vaginal bleeding” (Figure 1b).

When surveyed about danger signs during the postpartum period, 67% of women responded “*agree*” or “*strongly agree*” when asked about sufficiency of knowledge; yet, the median number of signs offered was 1 (IQR (0–2)) out of 9 possible. The most mentioned postpartum signs among women were “vaginal bleeding” and “abdominal pain” (Figure 1c).

**Figure 2:**
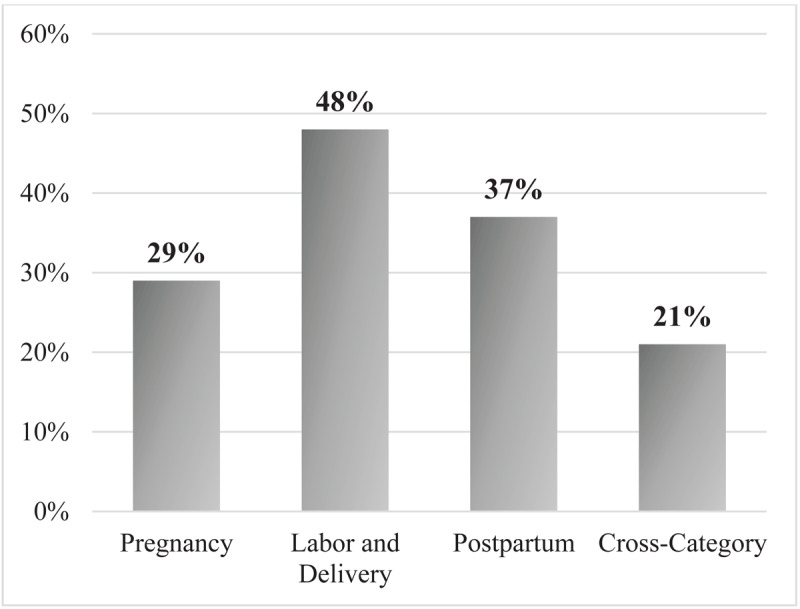
Percentage of women who could not spontaneously recall *any* danger signs.

**Figure 3:**
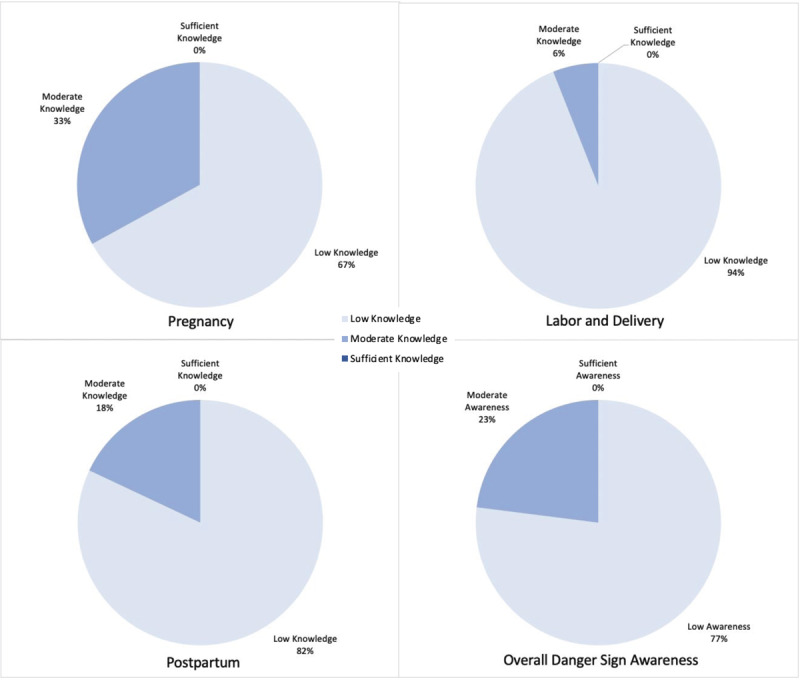
Knowledge of danger signs during **a)** pregnancy (n = 100); b) labor and delivery (n = 100); and **c)** postpartum (n = 100), and **d)** Overall cross-category danger sign awareness of surveyed women (n = 100).

Surveyed women were able to list a median of 4 danger signs (IQR (1–6)) out of 28 total. Figure 2 depicts the number of women unable to list *any* pregnancy, labor and delivery, postpartum, and summative cross-category danger signs, which was up to 48% of respondents. The overall knowledge levels for pregnancy, labor and delivery, and postpartum danger sign recall as well as overall awareness scores for cross-category recall are depicted in Figures 3a–d. When danger sign recall was trichotomized, 0% of respondents showed sufficient knowledge of danger signs across individual surveys for pregnancy, labor and delivery, and postpartum (Figures 3a–c). In continuation, when an overall awareness score was generated based on total recall, 0% of respondents showed sufficient awareness for all danger signs (Figure 3d).

Additionally, 94% of women in the study reported willingness to seek proper medical attention upon recognition of these signs. Of those, 78% reported a preference to visit a hospital and 59% reported a preference to visit a health center over the other options provided (traditional birth attendant, traditional healer, stay at home, or other).

### Knowledge scores and sociodemographic factors

We noted a significant relationship between highest level of education and overall number of danger signs recalled (out of 28 total) as well as overall graded awareness level (low, moderate, or sufficient). Individuals who completed higher levels of education were able to recall more danger signs and had better awareness scores as compared with individuals who did not read or write (Table 4). Those who had completed high school or Arabic school and college, professional, or graduate school were more likely to have higher knowledge of pregnancy-related danger signs (Table 5). Additionally, monthly income was correlated with increased overall awareness of obstetric danger signs (Table 4). Participants who had a monthly income of more than D 5,000 were more likely to have higher pregnancy-related danger sign knowledge and higher overall awareness (Table 5). Gravidity was not significantly associated with greater number of danger signs recalled nor overall awareness (Table 4), though those who had been pregnant two or more times were more likely to have a higher overall awareness score (Table 5).

**Table 4:** Sociodemographic factors associated with cross-category knowledge of danger signs and overall awareness score.


		n	p VALUE (OVERALL DANGER SIGN RECALL)	SPEARMAN RANK COEFFICIENT (ρ) (OVERALL DANGER SIGN RECALL)	p VALUE (OVERALL AWARENESS LEVEL)	SPEARMAN RANK COEFFICIENT (ρ) (OVERALL AWARENESS LEVEL)

**Age**	<18	0	0.179	0.136	0.198	0.123

	18–24	25				

	25–29	28				

	30–39	39				

	40–49	8				

	>50	0				

**Education Level**	Do not read or write	13	0.015*	0.243	0.008*	0.265

	Less than high school	33				

	Completed high school/Arabic School	37				

	College/Professional School/Graduate School	17				

**Monthly Income**	Less than D 5,000	27	0.136	0.151	0.002*	0.311

	D ≥ 5,000	72				

**Gravidity**	G1	19	0.316	0.101	0.615	0.051

	G2	13				

	G3+	68				


* Significant values indicated by boldface type and asterisk. Some categories were combined for logistical regression based on category size and observed outcomes.

**Table 5:** Odds of having awareness based on sociodemographic factors.


		n	OR (95% CI) FOR KNOWLEDGE ABOUT DANGER SIGNS OF PREGNANCY	OR (95% CI) FOR KNOWLEDGE ABOUT DANGER SIGNS OF LABOR & DELIVERY	OR (95% CI) FOR KNOWLEDGE ABOUT DANGER SIGNS OF POSTPARTUM	OR (95% CI) FOR OVERALL KNOWLEDGE ABOUT DANGER SIGNS

**Age**	<18	0	1.0	1.0	1.0	1.0

	18–24	25	**	**	**	**

	25–29	28	**	**	**	**

	30–39	39	**	**	**	**

	40–49	8	**	**	**	**

	>50	0	N/A	N/A	N/A	N/A

**Education Level**	Do not read or write	13	1.0	1.0	1.0	1.0

	Less than high school	33	3.84 (0.60, 75.46)	**	**	1.66 (0.22, 34.17)^†^

	Completed high school/Arabic school	37	9.14 (1.55, 175.35)*	**	**	5.76 (0.96, 111.12)^†^

	College/Professional/Graduate School	17	10.67 (1.55, 217.04)*	**	**	6.55 (0.92, 134.21)^†^

**Monthly Income**	Less than D 5,000	27	1.0	1.0	1.0	1.0

	D ≥5,000	72	3.88 (1.32, 14.26)*	**	**	5.14 (1.36, 33.78)*

**Gravidity**	G1	19	1.0	1.0	1.0	1.0

	G2	13	13.60 (2.49, 112.83)*	**	**	**

	G3+	68	4.13 (1.03, 27.88)*	**	**	**


* Significant at p < 0.05. ^†^ Indicates almost significant tendency, p = 0.052. ** *P*-value of the variable was greater than 0.05 which was not a candidate for logistic regression. Some categories were combined for logistical regression analysis based on category size and observed outcomes.

### Technology usage and education preferences

Seventy-six percent (76%) of participants owned a smartphone and 75% reported everyday use. Most smartphones were Android (76%). Most participants (96%) reported familiarity with smartphone applications, but 88% were either not familiar with smartphone applications or had never used smartphone applications for pregnant women (Gifted Mom, MomConnect, Safe Delivery App, and Zero Mothers Die). When asked about their interest in using a messaging app such as “WhatsApp” to learn more about pregnancy and childbirth related danger signs, 97% of women responded “*agree*” or “*strongly* agree.”

**Figure 4:**
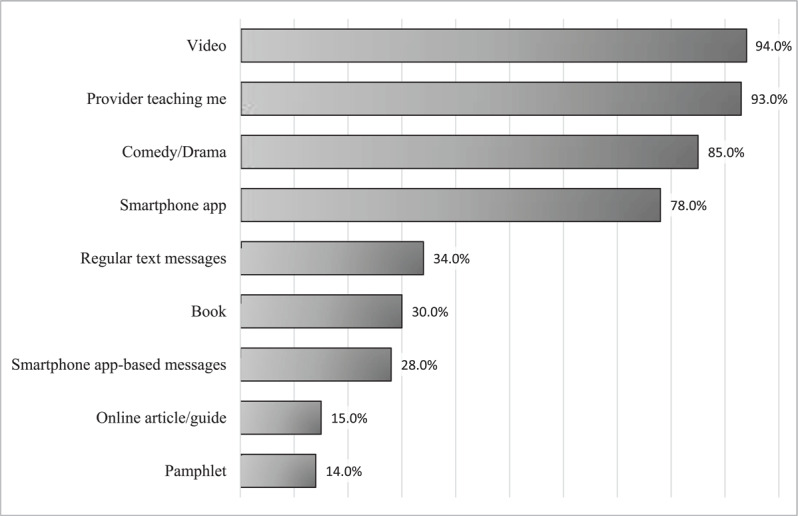
Danger sign education delivery method preferences among surveyed women.

The respondents’ preferences for educational platforms are represented in Figure 4. The most common preferences were “video” or “provider teaching me (nurse, midwife, doctor).”

Only 40% of participants reported access to a reliable internet connection and 69% reported reliable access to cellular data. Approximately half the participants (52%) reported ability to download smartphone applications using the internet.

## Discussion

Our findings indicate that overall knowledge of danger signs among women in The Gambia is low. No women in the study showed “sufficient” knowledge of danger signs (Figure 3), and up to 48% of respondents were unable to recall *any* signs for pregnancy, labor and delivery, or postpartum. Twenty-one respondents were unable to recall any danger signs across all categories (Figure 2). This observed pattern amongst women who participated in this study is not isolated. Similar findings have been documented in Tanzania [5,10], Uganda [15], and Ethiopia [6], where studies have shown between 19% and 41% of women were able to recall ≥2 danger signs per category. In addition, a study in India conducted by Agarwal et al. noted low complication readiness among mothers, suggesting that this low awareness of danger signs and understanding of instances in which emergency action is needed may be relatively universal among women in low-income countries.

**Figure 5:**
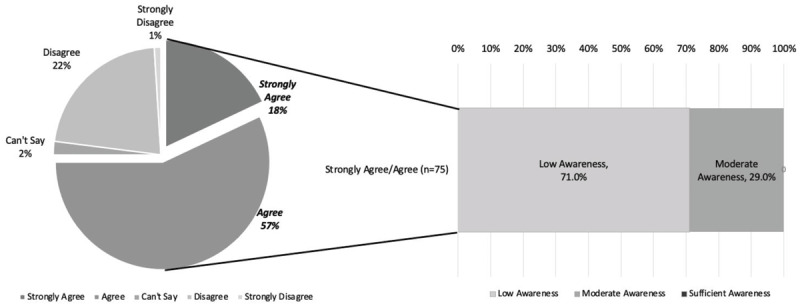
*Self-perception of danger sign awareness vs. reality of graded awareness on danger sign recall*. **(a.)** Breakdown of self-rating of “sufficient” awareness on Likert scale of question “I believe that I have sufficient knowledge of the danger signs that can occur during pregnancy.” **(b.)** Graded awareness for women who responded, “strongly agree” and “agree.”

A unique finding in our study was the stark difference between the pareicipants’ perceived awareness and fund of knowledge versus actual ability to spontaneously recall danger signs when prompted. Most participants (87%) stated that they had heard about complications related to pregnancy and 75% had a self-perceived “high” awareness of danger signs; yet, the median recall was only 1–2 signs and no woman in the study displayed sufficient knowledge (Figure 5).

Almost 80% of participants had been educated by their provider on maternal health, which raises the question of quality and content of education–are these women being adequately instructed on what physiologic signs to look for, what signs and symptoms indicate distress for either mom or fetus, and what actionable steps to take when these danger signs arise? Furthermore, it is concerning that several women in the study were only able to identify one danger sign per category, mostly “bleeding.” This is consistent with other studies performed in Tanzania [5], Uganda [15], and Ethiopia [6], in which “bleeding” was also most commonly mentioned. In our study, concerning signs such as “high fever” (which could indicate endometritis), and “blurry vision” and “headache” (which could be concerning signs of pre-eclampsia or eclampsia) were rarely mentioned (Figure 1). Kabakyenga et al. demonstrated similar findings, with only 1.6% of women in the Ugandan study able to mention “blurry vision” and 31% able to recognize “high fever” [15]. In Tanzania, Mwilike et al. found a higher proportion of women able to recognize “headache” as a danger sign (43.6%) [5], which may indicate educational or questionnaire design discrepancies. Regardless, more research is needed on the content of maternal obstetric education and provider counseling methods to understand the breadth of information and specific signs that may be overlooked when instructing mothers.

Our study found a significant relationship between education level and both total number of recalled danger signs and overall graded awareness level (Table 4). Similar studies in Ethiopia [23], Tanzania [10], and Kenya [24] found highest education level to be positively associated with ability to recall obstetric danger signs. This suggests that a greater level of education may influence understanding of explanations for health complications or encourage independent learning about pregnancy-related complications outside of routine obstetric visits. The relationship between education and health literacy has been well documented in low-, middle-, and high-income countries, with higher levels of educational attainment strongly linked to greater health literacy and health outcomes [25,26]. We also noted a significant association between monthly income and awareness score in this cohort, which we posit may also represent the well documented influence of lower socioeconomic status on health literacy [25,26].

Of note, our study found no significant relationship between age and awareness level of the participants. Prior studies in Tanzania [5] and Cameroon [27] found a positive association with age and level of awareness. This discrepancy may be due to regional factors in education or study size, as both studies used sample sizes of 384 and 532 participants, respectively. More investigation with larger patient sample size is needed to elucidate the relationship between age and awareness of danger signs in The Gambia.

We found that gravidity was not correlated with a higher number of danger signs recalled, but that women who had been pregnant over two times were more likely to have higher awareness levels. Prior studies in Cameroon [27] and Ethiopia [28] both found a positive association between gravidity and awareness of danger signs, with greater awareness in multiparous and grand-multiparous women. In these studies, the increased awareness was attributed to prior experience with pregnancy and ability to differentiate normal from abnormal, which we posit may be similarly observed here, though further studies with larger cohorts are needed to determine correlation and the nature of the relationship.

Finally, participants in this study almost universally demonstrated proper healthcare seeking behavior when danger signs arose, with 94% stating that they would go to a hospital or health center. This finding is promising for the efficacy of education, suggesting that augmentation of awareness would lead to greater proportion of mothers seeking care for identified emergencies and reduce preventable morbidities.

### Considerations for educational intervention

The findings of this study suggest that targeted educational interventions are needed to increase awareness of danger signs in women of childbearing age in The Gambia. Given the low literacy rate of women in the country, which was cited by UNESCO in 2015 to be 41.57% in adult females over the age of 18 [29], we infer that interventions that are video- or audio-based would be more accessible to a larger group of women. Women in our study preferred videos (94%) and provider teaching (93%). Here, surveyed women noted the largest barriers to care to be distance, referrals, and associated costs with travel to healthcare centers. Therefore, educational intervention that can be delivered remotely would be most accessible.

Additionally, patients noted a high level of trust in providers (94%), suggesting good patient rapport among nurses, midwives, and doctors. Most current obstetric education is provided via nurses and midwives, and we suggest that local providers could deliver teaching via a video-based recording. If women already trust these individuals and respect the ability of providers to deliver accurate information, videos by the providers themselves may be more well received by mothers-to-be.

Women also expressed a high interest in app-based content (78%) though given the relatively low rate of reliable internet connection (40%) and cellular data (69%), an app-based system may not be able to reach as many women in the local area. Additionally, our study only included one rural healthcare center, so accessibility to internet and data may be overrepresented by the largely urban-based participants. However, most women have smartphones or regular mobile phones, in which case delivery of educational videos via regular text messaging would remove the need for internet access. The majority of women in our study (97%) noted that they would be interested in messaging-based education, so this option would align with the preferences of the women that were included in our survey.

More investigation is needed into the best delivery method for educational content, especially in rural areas and those least accessible by internet or cellular data. Pilot studies and feedback from Gambian women will be essential to understand which content, media, and mode of delivery is most effective and accessible for as many women as possible.

### Future Directions

Further research is needed to understand the relationship between recognition of danger signs and sociodemographic factors that contribute to knowledge. In the future, greater sample size and more rural site assessment would be beneficial. In addition, study efforts could investigate if temporality of education contributes to a women’s ability to spontaneously recall danger signs. In our current study, women who were pregnant at least once in the last five years were eligible, but we did not tailor questions to investigate the relationship between time from last pregnancy and awareness of danger signs. This would have allowed us to better understand specifically when educational delivery and refresher education is needed for both the women and providers.

In addition to an assessment of obstetric patients, future studies should assess provider delivery methods and content of provider teaching. In this way, we will be better able to tailor educational methods to not only the women, but also those nurses, midwives, and physicians who are counseling during routine visits. Creating a content framework for counseling patients and coaching providers on delivery of obstetric education is a future goal of this project.

## Conclusions

To our knowledge, this is the first study to directly assess both danger sign awareness in Gambian women and attitudes and access towards technological education intervention. Our findings indicate that knowledge of obstetric danger signs among women in The Gambia is low. More importantly, self-perceived knowledge does not correlate with true ability to recognize signs. This may lead to dangerous false assumptions with women thinking they are prepared for complications but unable to recognize and seek emergent medical care when necessary. Women want to seek healthcare; they just need to know how to recognize *when* care is needed. Interventions aimed at teaching women about danger signs shows much promise, and augmenting awareness via education could lead to quicker action and a greater proportion of mothers seeking care for identifiable emergencies. The established patient rapport with providers in combination with utilization of technology by women suggest that local provider-recorded informational videos sent through text or app-based messaging would be both accessible and well received by mothers-to-be. Further studies are indicated for trial implementation and efficacy assessment of developed educational tools.

## References

[1] WHO, UNICEF, UNFPA, WBG, United Nations Population Division. Trends in Maternal Mortality 2000–2017; 2019.

[2] WHO. The World Health Report 2005: Make Every Mother and Child Count; 2005. 10.1080/14034940500217037 16332605

[3] Yaya S, Bishwajit G. Predictors of institutional delivery service utilization among women of reproductive age in Gambia: a cross-sectional analysis. BMC Pregnancy Childbirth. 2020; 20(1): 187. DOI: 10.1186/s12884-020-02881-4 32228501 PMC7106584

[4] Khan KS, Wojdyla D, Say L, Gulmezoglu AM, Van Look PF. WHO analysis of causes of maternal death: A systematic review. Lancet. 2006; 367(9516): 1066–1074. DOI: 10.1016/S0140-6736(06)68397-9 16581405

[5] Mwilike B, Nalwadda G, Kagawa M, Malima K, Mselle L, Horiuchi S. Knowledge of danger signs during pregnancy and subsequent healthcare seeking actions among women in Urban Tanzania: A cross-sectional study. BMC Pregnancy Childbirth. 2018; 18(4). DOI: 10.1186/s12884-017-1628-6 PMC5751870 29295710

[6] Hailu D, Hailemariam B. Knowledge about obstetric danger signs and associated factors among mothers in Tsegedie district, Tigray region, Ethiopia 2013: Community based cross-sectional study. PLoS ONE. Published online February 6, 2014. DOI: 10.1371/journal.pone.0083459 PMC3916287 24516516

[7] Woldeamanuel GG, Lemma G, Zegeye B. Knowledge of obstetric danger signs and its associated factors among pregnant women in Angolela Tera District, Northern Ethiopia. BMC Res Notes. 2019; 12(1): 606. DOI: 10.1186/s13104-019-4639-8 31547838 PMC6755683

[8] Nkamba DM, Wembodinga G, Bernard P, Ditekemena J, Robert A. Awareness of obstetric danger signs among pregnant women in the Democratic Republic of Congo: Evidence from a nationwide cross-sectional study. BMC Womens Health. 2021; 21: 82. DOI: 10.1186/s12905-021-01234-3 33637065 PMC7908745

[9] Geleto A, Chojenta C, Abdulbasit M, Loxton D. WOMEN’s knowledge of obstetric danger signs in Ethiopia (WOMEN’s KODE): A systematic review and meta-analysis. Syst Rev. 2019; 8(63). DOI: 10.1186/s13643-019-0979-7 PMC6388496 30803443

[10] Pembe AB, Urassa DP, Carlstedt A, Lindmark G, Nyström L, Darj E. Rural Tanzanian women’s awareness of danger signs of obstetric complications. BMC Pregnancy Childbirth. 2009; 9(1): 12. DOI: 10.1186/1471-2393-9-12 19323836 PMC2667432

[11] Aborigo RA, Moyer CA, Gupta M, et al. Obstetric danger signs and factors affecting health seeking behaviour among the Kassena-Nankani of Northern Ghana: a qualitative study. Afr J Reprod Health. 2014; 18(3): 78–86. 25438512

[12] Regasa MT, Markos J, Habte A, Upashe SP. Obstetric danger signs: knowledge, attitude, health-seeking action, and associated factors among postnatal mothers in Nekemte town, Oromia region, Western Ethiopia—A community-based cross-sectional study. Obstet Gynecol Int. 2020; 6573153. DOI: 10.1155/2020/6573153 32952565 PMC7481917

[13] Cham M, Sundby J, Vangen S. Maternal mortality in the rural Gambia, a qualitative study on access to emergency obstetric care. Reprod Health. 2005; 2(1): 3. DOI: 10.1186/1742-4755-2-3 15871743 PMC1142340

[14] Coleman A. The use of ICT tools (mobile phones) to improve awareness of pregnancy danger signs among pregnant women in rural communities of South Africa. Published online 2014. Accessed May 30, 2021. /paper/The-Use-of-ICT-Tools-(Mobile-Phones)-to-Improve-of-Coleman/f819e433f5f12224bd927c31008f06f711e39d3f.

[15] Kabakyenga JK, Ostergren PO, Turyakira E, Pettersson KO. Knowledge of obstetric danger signs and birth preparedness practices among women in rural Uganda. Reprod Health. 2011; 8: 33. DOI: 10.1186/1742-4755-8-33 22087791 PMC3231972

[16] Kunetsor S, Walley J, Katabira E, et al. Using mobile phones to improve clinic attendance amongst an antiretroviral treatment cohort in rural Uganda: A cross-sectional and prospective study. AIDS Behav. 2014; 14(6): 1347–1352. DOI: 10.1007/s10461-010-9780-2 20700644

[17] Musiimenta A, Tumuhimbise W, Mugyenyi G, Katusiime J, Atukunda EC, Pinkwart N. Mobile phone-based multimedia application could improve maternal health in rural southwestern Uganda: Mixed methods study. Online J Public Health Inform. 2020; 12(1): e8. DOI: 10.5210/ojphi.v12i1.10557 32742558 PMC7386055

[18] Gambia Population 2021 (Demographics, Maps, Graphs). Accessed December 30, 2021. https://worldpopulationreview.com/countries/gambia-population.

[19] JHPIEGO. Monitoring birth preparedness and complication readiness: Tools and indicators for maternal and newborn health. JHPIEGO. Published online 2004: 338.

[20] WHO. Pregnancy, Childbirth, postpartum and newborn care: A guide for essential practice. 3rd ed. 2015. Accessed December 30, 2021. http://www.ncbi.nlm.nih.gov/books/NBK326678/. 26561684

[21] The Gambia minimum wage – World minimum wage rates 2022. Accessed July 26, 2022. https://www.minimum-wage.org/international/the-gambia.

[22] Markets Insider. Dalasi to US-Dollar conversion | GMD to USD exchange rate calculator. Accessed December 30, 2021. https://markets.businessinsider.com/currency-converter/gambian-dalasi_united-states-dollar.

[23] Bililign N, Mulatu T. Knowledge of obstetric danger signs and associated factors among reproductive age women in Raya Kobo district of Ethiopia: A community based cross-sectional study. BMC Pregnancy Childbirth. 2017; 17(70). DOI: 10.1186/s12884-017-1253-4 PMC5320700 28222694

[24] Mutiso SM, Qureshi Z, Kinuthia J. Birth preparedness among antenatal clients. East Afr Med J. 2008; 85(6): 275–283. DOI: 10.4314/eamj.v85i6.9625 18817024

[25] Kickbusch I, Pelikan JM, Apfel F, Tsouros AD, WHO, (eds.) Health Literacy: The Solid Facts. World Health Organization Regional Office for Europe; 2013.

[26] Schillinger D. The intersections between social determinants of health, health literacy, and health disparities. Stud Health Technol Inform. 2020; 269: 22–41. DOI: 10.3233/SHTI200020 32593981 PMC7710382

[27] Emeh AN, Atem AN, Humphrey AA, Gilbert TN, Landis FC. Antenatal care and determinants of obstetric danger signs awareness of immediate postpartum women at Buea Regional Hospital, Cameroon. Pan Afr Med J. 2021; 38: 247. DOI: 10.11604/pamj.2021.38.247.20977 34104295 PMC8164428

[28] Maseresha N, Woldemichael K, Dube L. Knowledge of obstetric danger signs and associated factors among pregnant women in Erer district, Somali region, Ethiopia. BMC Womens Health. 2016; 16(1): 30. DOI: 10.1186/s12905-016-0309-3 27265154 PMC4893837

[29] Literacy rate, adult female (% of females ages 15 and above) – The Gambia | Data. Accessed January 4, 2022. https://data.worldbank.org/indicator/SE.ADT.LITR.FE.ZS?locations=GM.

